# Effects of paroxetine hydrochloride combined with idebenone on inflammatory factors and antioxidant molecules in treatment of depression after ischemic stroke

**DOI:** 10.12669/pjms.39.1.5679

**Published:** 2023

**Authors:** Li-li Yuan, Tian-yu Chen, Zhi-qiang Huang

**Affiliations:** 1 Li-li Yuan, Xingtai Sanli Health Quannan Clinic, Xingtai 054001, Hebei, P. R. China; 2 Tian-yu Chen, Applied Psychology Major of Shijiazhuang University, Shijiazhuang 050024, Hebei, P. R. China; 3 Zhi-qiang Huang Department of Neurology, The First Affiliated Hospital of Xingtai Medical College, Xingtai 054001, Hebei, P. R. China

**Keywords:** Paroxetine hydrochloride, Idebenone, Depression after ischemic stroke, Treatment

## Abstract

**Objectives:**

To evaluate the effects of paroxetine hydrochloride combined with idebenone on inflammatory factors and antioxidant molecules in the treatment of depression after ischemic stroke.

**Methods:**

Randomized controlled trial was adopted on 80 patients with depression after ischemic stroke were randomly divided into two groups, with 40 patients in each group at Xingtai Sanli Health Quannan Clinic from March 17, 2019 to December 20, 2021. Both groups were given basic treatment. On this basis, the control group was treated with paroxetine hydrochloride, while the study group was treated with paroxetine hydrochloride combined with idebenone. The clinical efficacy was evaluated using the Hamilton Rating Scale for Depression (HRSD) before and after treatment. Additionally, the difference in HRSD score after treatment and the improvement in inflammatory factors and antioxidant molecules were compared and analyzed between the two groups.

**Results:**

After treatment, the HRSD score of the study group was significantly improved compared with that of the control group (*p=* 0.00). The effective rate was 82.5% in the study group, which was significantly higher than 62.5% in the control group (*p=* 0.04). After treatment, TNF-a, CRP and IL-6 in the study group were significantly lower than those in the control group (*p*= 0.00). Serum SOD, TAC and CAT levels in the study group were significantly higher than those in the control group after treatment (SOD and TAC, *p=* 0.00; CAT, *p=* 0.01). The incidence of adverse reactions was 37.5% in the study group and 25% in the control group. Although the incidence of adverse reactions in the study group was higher than that in the control group, the difference was not statistically significant (*p=* 0.23).

**Conclusion:**

Paroxetine hydrochloride combined with idebenone in the treatment of depression after ischemic stroke can significantly improve HRSD score, enhance clinical efficacy, reduce the levels of inflammatory factors, and increase the levels of antioxidant factors, without a significant increase in adverse reactions. Therefore, it is a safe and effective treatment method.

## INTRODUCTION

Ischemic stroke is a clinically common cerebral circulatory disorder in the middle-aged and elderly,^1^ with high incidence, disability rate and mortality. Ischemic stroke leads to a variety of pathophysiological abnormalities, including brain inflammation, neuronal loss, cognitive impairment and depression.^2^ Post-stroke depression (PSD) is a common complication in stroke patients, which seriously affects the rehabilitation of neurological function and the quality of life. Clinically, it is also called vascular depression, which causes great harm to patients. Patients will present physical discomfort, insomnia, dreaminess and depression, and some will have suicidal thoughts, even leading to suicide.^3^ Therefore, actively treating patients with PSD and effectively controlling their depressive symptoms are of great clinical significance to promote their physical and mental health and improve their quality of life.^4^ At present, PSD is mainly treated with antidepressants in clinic. However, in long-term clinical practice, it is found that antidepressant therapy alone in the treatment of PSD can not achieve ideal clinical efficacy.^5^ A current study has reported that for patients with PSD, on the basis of routine antidepressant therapy, the combined use of brain-protective agents can significantly improve the clinical efficacy.^6^ In this study, depression after ischemic stroke was treated using paroxetine hydrochloride combined with idebenone, and good results were achieved.

## METHODS

This randomized controlled trial was adopted on 80 patients with depression after ischemic stroke were randomly divided into two groups, with 40 patients in each group at Xingtai Sanli Health Quannan Clinic from March 17, 2019 to December 20, 2021. There were 24 males and 16 females in the study group, aged 56~75 years (average, 67.58 ± 5.47 years), and 23 males and 17 females in the control group, aged 55~74 years (average, 66.75 ± 5.84). No significant differences were found in the general data between the two groups, suggesting comparability (Table-I).

**Table-I:** Comparison of general data between study group and control group (*χ̅*± *S*) n = 40.

Index	Study group	Control group	t/χ^2^	P
Age (year)	67.58 ± 5.47	66.75 ± 5.84	0.66	0.51
Male (n %)	24 (60%)	23 (57.5%)	0.05	0.82
***Educational level***				
Primary school	14 (35%)	15 (37.5%)	0.05	0.82
Middle school	15 (37.5%)	11 (27.5%)	0.91	0.34
University and above	11 (27.5%)	14 (35%)	0.52	0.47
***Past medical history***				
Hypertension	22 (55%)	26 (65%)	0.83	0.36
Diabetes	13 (32.5%)	17 (42.5%)	0.85	0.36
Smoking history	19 (47.5%)	16 (40%)	0.45	0.50
History of alcoholism	12 (30%)	14 (35%)	0.23	0.67
HRSD score	28.72 ± 3.08	29.10 ± 3.74	0.50	0.62

*P* > 0.05

### Inclusion criteria:

- Patients below 75 years old;
- All patients with initial onset meeting the diagnostic criteria of ischemic stroke^7^;
- vPatients with ischemic stroke confirmed by head CT and/or MRI;
- Patients with the Hamilton Rating Scale for Depression-24 item (HRSD-24) score ≥ 20^8^;
- Patient with no obvious disturbance of consciousness and ability to cooperate with the research work;
- Patients without recent use of drugs affecting the study, such as immunosuppressants and hormone drugs;
- Patients and their families signing the consent form and having the ability to cooperate with the research work.

### Exclusion criteria:

- Patients with severe dementia, aphasia, disturbance of consciousness, cognitive impairment, or inability to cooperate with the researcher;
- Patients with depression before stroke;
- Patients with metabolic diseases or chronic consumptive diseases, such as tumors and chronic inflammatory diseases;
- Patients complicated with other mental diseases;
- Patients with allergy, intolerance or contraindications to the relevant drugs involved in the study;
- Patients with infectious diseases such as tuberculosis and hepatitis or complicated with important organ dysfunction, such as hepatic and renal dysfunction.

Both groups were given basic treatment, such as reducing blood lipid, controlling blood pressure, controlling blood glucose level, scavenging oxygen free radicals, nutritional support, nourishing brain cells and other symptomatic treatment. Additionally, the control group was treated with paroxetine hydrochloride alone, 20 mg/time, once a day. After administration for 2-3 weeks, it was increased by 10mg every week according to the patients’ response, with the maximum daily dose reaching 50mg. The study group was treated with paroxetine hydrochloride combined with Idebenone. The specific scheme: the usage of paroxetine hydrochloride was the same as that in the control group, and Idebenone 30mg, three times/day. The hepatic and renal functions of the two groups were reexamined every month.

### Observation Indexes:

(1) The improvement in depressive symptoms was evaluated using the HRSD score, and the score 12 weeks after treatment was compared and analyzed between the two groups. (2) Evaluation of clinical efficacy: After treatment for 12 weeks, the reduction rate of the HRSD score was used as the evaluation criterion.^9^ It was evaluated as cured: The HRSD score reduced by > 75% compared with that before treatment; significantly improved: HRSD score reduced by 50%~75% compared with that before treatment; improved: HRSD score reduced by 25%~50% compared with that before treatment; ineffective: HRSD score reduced by < 25%. Total effective rate = cured rate + significantly improved rate + improved rate. (3) Improvement in inflammatory factors: Peripheral venous blood (5ml) was collected from all patients in the morning before treatment and four weeks after treatment. The levels of inflammatory factors such as tumor necrosis factor-a (TNF-a), C-reactive protein (CRP) and interleukin-6 (IL-6) were detected using enzyme-linked immunosorbent assay (ELISA). (4) Improvement in antioxidant molecules: Peripheral venous blood (5 ml) was collected in the morning before treatment and four weeks after treatment, respectively. The changes in antioxidant molecules such as superoxide dismutase (SOD), glutathione peroxidase (GSH-Px), total antioxidant capacity (TAC), catalase (CAT) and glutathione reductase (GR) were determined by radioimmunoassay. (5) Evaluation of adverse drug reactions: Adverse drug reactions were recorded within four weeks after treatment, including rashes, gastrointestinal reactions, oral mucositis, white blood cell (WBC) reduction, neuritis and hepatic injury.

### Ethics Approval:

The study was approved by the Institutional Ethics Committee of Xingtai Sanli Health Quannan Clinic (No.:2020ZC282; Date: May 10, 2021), and written informed consent was obtained from all participants.

### Statistical Analysis:

All data were statistically analyzed using SPSS 20.0. The measurement data were expressed as (*χ̄*±*S*). Inter-group data were analyzed by the two independent sample t-test, intra-group data were analyzed with the repeated measurement analysis of variance, and rates were compared using the χ^2^ test. *P* < 0.05 was considered as statistically significant.

## RESULTS

The improvement of depressive symptoms in the two groups is shown in Table-II. The HRSD scores of the study group and the control group were > 20 before treatment, without statistically significant difference (*p=* 0.62). After treatment, the HRSD score of the study group was significantly improved than that of the control group (*p=* 0.00).

**Table-II:** Comparison of improvement in depressive symptoms between the two groups (*χ̅*± *S*) n = 40.

Group	Before treatment	After treatment*
Study group	28.72 ± 3.08	11.29 ± 5.47
Control group	29.10 ± 3.74	18.76 ± 6.34
t	0.50	5.64
P	0.62	0.00

* *P* < 0.05

The analysis of the two groups showed that the effective rate was 82.5% in the study group and 62.5% in the control group. The effective rate in the study group was significantly higher than that in the control group (*p=* 0.04, Table-III).

**Table-III:** Comparison of clinical efficacy between the two groups (*χ̅*±*S*) n=40.

Group	Cured	Significantly improved	Improved	Ineffective	Effective rate
Study group	10	11	12	7	33 (82.5%)
Control group	7	10	8	15	25 (62.5%)
χ^2^					4.01
P					0.04

*P* < 0.05

Before treatment, no significant differences were found in TNF-a, CRP and IL-6 between the study group and the control group (*P* > 0.05). After treatment, TNF-a, CRP and IL-6 in the study group were significantly lower than those in the control group (*p=* 0.00) (Table-IV).

**Table-IV:** Comparison of changes in inflammatory factors between the two groups before and after treatment (*χ̅*±*S*) n=40.

Index		Study group	Control group	t	P
TNF-ɑ (ng/L)	Before treatment	43.75 ± 12.53	43.62 ± 12.74	0.05	0.96
	After treatment*	7.49 ± 2.08	10.41 ± 2.37	5.86	0.00
CRP (mg/L)	Before treatment	42.85 ± 8.62	43.51 ± 7.57	0.36	0.72
	After treatment*	6.97 ± 2.01	9.70 ± 2.24	5.74	0.00
IL-6 (ng/L)	Before treatment	9.28 ± 1.76	9.35 ± 1.65	0.18	0.85
	After treatment*	3.25 ± 0.59	5.71 ± 1.06	12.82	0.00

* *P* < 0.05

The comparative analysis of antioxidant molecules between the study group and the control group (Table-V) suggested that serum SOD, TAC and CAT levels in the study group were significantly higher than those in the control group after treatment (SOD and TAC, *p=* 0.00; CAT, *p=* 0.01). However, GSH-Px and GR levels presented no significant differences between the two groups (*P* > 0.05).

**Table-V:** Comparison of antioxidant molecules between study group and control group (U/ml, n=40,*χ̅* ± *S*).

Index		Study group	Control group	t	P
SOD	Before treatment	63.27 ± 7.52	63.62 ± 7.76	0.20	0.84
	After treatment*	68.92 ± 7.63	64.17 ± 6.85	2.93	0.00
GSH-Px	Before treatment	321.18 ± 22.15	325.64 ± 23.43	0.79	0.43
	After treatment	335.32 ± 25.57	334.63 ± 26.25	0.18	0.86
TAC	Before treatment	10.83 ± 2.01	10.77 ± 2.13	0.13	0.90
	After treatment*	15.74 ± 2.25	13.07 ± 3.12	4.38	0.00
CAT	Before treatment	8.46 ± 2.12	8.41 ± 2.39	0.09	0.92
	After treatment*	12.92 ± 4.16	10.58 ± 3.87	2.60	0.01
GR	Before treatment	122.35 ± 22.82	123.63 ± 21.07	0.21	0.84
	After treatment	127.71 ± 17.36	125.63 ± 16.12	0.54	0.58

* *P*< 0.05

Comparative analysis of adverse drug reactions between the two groups after treatment demonstrated that the incidence of adverse reactions was 37.5% in the study group and 25% in the control group. Although the incidence of adverse reactions in the study group was higher than that in the control group, the difference was not statistically significant (*p=* 0.23) (Table-VI).

**Table-VI:** Comparative analysis of adverse drug reactions between the two groups after treatment (*χ̅*± *S*) n = 40.

Group	Rashes	Gastrointestinal reactions	WBC reduction	Neural response	Hepatic injury	Incidence
Study group	2	4	3	3	3	15 (37.5%)
Control group	1	3	2	1	3	10 (25%)
*χ* ^2^						1.45
*P*						0.23

*P* > 0.05.

## DISCUSSION

Our study finally confirmed that the HRSD score of patients with depression after ischemic stroke treated with paroxetine hydrochloride combined with Idebenone was significantly improved compared with that of patients treated with paroxetine hydrochloride alone (*p=* 0.00), and the effective rate was significantly improved (*p=* 0.04). After treatment, TNF-a, CRP and IL-6 in the study group were significantly lower than those in the control group (*p=* 0.00), and the levels of serum SOD, TAC and CAT in the study group were significantly higher than those in the control group (SOD and TAC, *p=* 0.00; CAT, *p=* 0.01). The incidence of adverse reactions was 37.5% in the study group and 25% in the control group. Although the incidence of adverse reactions in the study group was higher than that in the control group, the difference was not statistically significant (*p=* 0.23).

Relevant clinical data^10^ show that patients with ischemic stroke are very prone to depression after onset, characterized by low spirits, lack of interest and sense of worthlessness, which are mostly accompanied by sleep disorders, cognitive defects and physical symptoms. The meta-analysis found that the incidence of depression two weeks - seven years after stroke was as high as 33%.^11^ PSD can not only lead to the disorders of neurological recovery and the loss of independent living ability, but also induce the recurrence of stroke and increase mortality. Generally speaking, patients with mild PSD mostly present reduced interest, insomnia, inattention, irritability, etc. With the aggravation of the disease, patients may have a loss of appetite and even hallucinations and suicidal tendencies. In addition, some patients may also be accompanied by atypical characteristics such as dizziness, chest tightness, nausea, vomiting and fatigue. Most patients do not present depressive symptoms immediately after stroke, but several months or even 2~3 years after stroke. Due to its occult onset, PSD is difficult to be diagnosed early and intervened timely.^12^

At present, the main pathogenesis of stroke complicated with depression are as follows: (1) Cytokines in the brain mainly affect the concentration and renewal of monoamine neurotransmitters in synapses, or affect the number and function of monoamine receptors through monoamine neurotransmitters (such as norepinephrine and 5-serotonin), thus reducing the function of monoamine transmitters.^13^ (2)Cytokines may activate the hypothalamic-pituitary-adrenal axis, corticotropin-releasing hormone and sympathetic hyperactivity, resulting in corresponding emotional and behavioral changes.^14^ Antidepressants are widely used in the treatment of this disease.^15^ Paroxetine hydrochloride is a selective norepinephrine reuptake inhibitor, which plays an important role in the clinical treatment of depression.^16^ Paroxetine hydrochloride plays an antidepressant role mainly through the following mechanisms. On the one hand, it can selectively inhibit the reuptake of 5-serotonin by presynaptic neurons, so as to improve the concentration of 5-serotonin in the synaptic space. Moreover, the information transmission and motor improvement of 5-serotonin can improve the brain injury of patients.^17^ On the other hand, paroxetine hydrochloride can improve patients’ depression, and plays an important role in improving patients’ subjective initiative.^18^

For patients with PSD, the simple application of antidepressants cannot obtain ideal efficacy. Studies^19^ have demonstrated that abnormal levels of inflammatory factors and antioxidant molecules play a very important role in the occurrence and progression of PSD. Waisman et al.^20^ Believe that a high IL-17 level is related to the degree of depression. PSD is a common complication of several inflammatory diseases and different types of central nervous system infection. The animal experiment results of Chen et al.^21^ Showed that the level of IL-6 was positively correlated with the increase of depression and anxiety-like behaviors after stroke in mice. Swardfager et al.^22^ Believe that the abnormalities of the central nervous system such as major depression (MDD) and bipolar disorder (BD) are related to inflammatios. The study by Verma suggested that the abnormalities of inflammatory factors, antioxidant molecules and immune cells could lead to neuronal loss, infiltration of microglia and infiltrating monocytes/macrophages in the intercellular matrix, and aggravate or induce PSD. Ułamek et al^24^ study confirmed that the potential mechanisms of tau protein in the brain after ischemia, including a series of pathophysiological reactions such as oxidative stress, apoptosis, autophagy, excitotoxicity, neuroinflammation, endothelium, angiogenesis and mitochondrial dysfunction, were involved in the progression of PSD. Additionally, the study of Carboni et al.^25^ Confirmed that TNF-α, IL-6, IL-10 and CRP levels were correlated with reduced severity of depression. The potentials of IL-10, IL-6 and TNF-α, as biomarkers of broader antidepressant response deserve further study. Idebenone is an effective antioxidant and CoQ10 analogue.^26^ It has been shown that the anti-inflammatory effect of NLRP3-mediated injury in I/R can be improved by inhibiting the activation of NLRP3, which may provide new insights into the treatment strategy of ischemic stroke.^27^ Reducing oxidative stress and improving brain inflammation can play a neuroprotective role and treat PSD.^28^

### Limitations:

It includes small sample size and short follow-up time. In our future work, we will enlarge the sample size and increase follow-up time, with the expectation of elaborating the long-term effect and benefit of this treatment scheme to patients.

## CONCLUSION

In conclusion, paroxetine hydrochloride combined with Idebenone in the treatment of depression after ischemic stroke can significantly improve HRSD score, enhance clinical efficacy, reduce the levels of inflammatory factors, and increase the levels of antioxidant factors, without a significant increase in adverse reactions. Therefore, it is a safe and effective treatment method.

### Authors’ Contributions:

**LLY:** Designed this study and prepared this manuscript, are responsible and accountable for the accuracy and integrity of the work.

**TYC:** Collected and analyzed clinical data.

**ZQH:** Data analysis, significantly revised this manuscript.

## References

[1] Wang X, Sun Y, Dong S, Liu X, Ji J (2019). Butyphthalide in the treatment of massive Cerebral Infarction. Pak J Med Sci.

[2] Maheshwari AK, Kumar P, Alam MT, Aurangzeb M, Parkash J, Imran K (2016). Frequency of Hyperthermia in Acute Ischemic Stroke Patients Visiting a Tertiary Care Hospital. J Coll Physicians Surg Pak.

[3] Shishkina GT, Kalinina TS, Gulyaeva NV, Lanshakov DA, Dygalo NN (2021). Changes in Gene Expression and Neuroinflammation in the Hippocampus after Focal Brain Ischemia:Involvement in the Long-Term Cognitive and Mental Disorders. Biochemistry (Mosc).

[4] Syafrita Y, Amir D, Susanti R, Fadhilah I (2020). Relationship of brain-derived neurotrophic factor, malondialdehyde, and 8-Hydroxy 2-Deoxyguanosine with post-ischemic stroke depression. Dement Neuropsychol.

[5] Li L, Han Z, Li L, Han L, Yan B (2020). Effectiveness of Paroxetine for Poststroke Depression:A Meta-Analysis. J Stroke Cerebrovasc Dis.

[6] Yan A, Liu Z, Song L, Wang X, Zhang Y, Wu N (2019). Idebenone Alleviates Neuroinflammation and Modulates Microglial Polarization in LPS-Stimulated BV2 Cells and MPTP-Induced Parkinson's Disease Mice. Front Cell Neurosci.

[7] Takahashi M, Hashimoto M, Uehara M (2018). Nihon Hoshasen Gijutsu Gakkai Zasshi.

[8] Parker G, Hadzi-Pavlovic D (2020). Do Hamilton depression scale items have the capacity to differentiate melancholic and non-melancholic depressive sub-types?. J Affect Disord.

[9] Rosso IM, Killgore WD, Olson EA, Webb CA, Fukunaga R, Auerbach RP (2017). Internet-based cognitive behavior therapy for major depressive disorder:A randomized controlled trial. Depress Anxiety.

[10] Song J, Kim OY (2016). Galanin's implications for post-stroke improvement. Anat Cell Biol.

[11] Mitchell AJ, Sheth B, Gill J, Yadegarfar M, Stubbs B, Yadegarfar M (2017). Prevalence and predictors of post-stroke mood disorders:A meta-analysis and meta-regression of depression, anxiety and adjustment disorder. Gen Hosp Psychiatry.

[12] Medeiros GC, Roy D, Kontos N, Beach SR (2020). Post-stroke depression:A 2020 updated review. Gen Hosp Psychiatry.

[13] Zhang E, Liao P (2020). Brain-derived neurotrophic factor and post-stroke depression. J Neurosci Res.

[14] Menard C, Hodes GE, Russo SJ (2016). Pathogenesis of depression:Insights from human and rodent studies. Neuroscience.

[15] Das J, Rajanikant GK (2018). Post stroke depression:The sequelae of cerebral stroke. Neurosci Biobehav Rev.

[16] Cipriani A, Furukawa TA, Salanti G, Chaimani A, Atkinson LZ, Ogawa Y (2018). Comparative efficacy and acceptability of 21 antidepressant drugs for the acute treatment of adults with major depressive disorder:A systematic review and network meta-analysis. Lancet.

[17] Borrelli J, Starr A, Downs DL, North CS (2019). Prospective Study of the Effectiveness of Paroxetine on the Onset of Posttraumatic Stress Disorder, Depression, and Health and Functional Outcomes After Trauma. J Orthop Trauma.

[18] Sagarwala R, Nasrallah HA (2019). Changes in inflammatory biomarkers before and after SSRI therapy in PTSD:A review. Ann Clin Psychiatry.

[19] Meng G, Ma X, Li L, Tan Y, Liu X, Liu X (2017). Predictors of early-onset post-ischemic stroke depression:a cross-sectional study. BMC Neurol.

[20] Waisman A, Hauptmann J, Regen T (2015). The role of IL-17 in CNS diseases. Acta Neuropathol.

[21] Chen JY, Yu Y, Yuan Y, Zhang YJ, Fan XP, Yuan SY (2017). Enriched housing promotes post-stroke functional recovery through astrocytic HMGB1-IL-6-mediated angiogenesis. Cell Death Discov.

[22] Swardfager W, Hennebelle M, Yu D, Hammock BD, Levitt AJ, Hashimoto K (2018). Metabolic/inflammatory/vascular comorbidity in psychiatric disorders;soluble epoxide hydrolase (sEH) as a possible new target. Neurosci Biobehav Rev.

[23] Verma R, Cronin CG, Hudobenko J, Venna VR, McCullough LD, Liang BT (2017). Deletion of the P2X4 receptor is neuroprotective acutely, but induces a depressive phenotype during recovery from ischemic stroke. Brain Behav Immun.

[24] Ułamek-Kozioł M, Czuczwar SJ, Januszewski S, Pluta R (2020). Proteomic and Genomic Changes in Tau Protein, Which Are Associated with Alzheimer's Disease after Ischemia-Reperfusion Brain Injury. Int J Mol Sci.

[25] Carboni L, McCarthy DJ, Delafont B, Filosi M, Ivanchenko E, Ratti E (2019). Biomarkers for response in major depression:comparing paroxetine and venlafaxine from two randomised placebo-controlled clinical studies. Transl Psychiatry.

[26] Qi FX, Hu Y, Kang LJ, Li P, Gao TC, Zhang X (2020). Effects of Butyphthalide Combined with Idebenone on Inflammatory Cytokines and Vascular Endothelial Functions of Patients with Vascular Dementia. J Coll Physicians Surg Pak.

[27] Peng J, Wang H, Gong Z, Li X, He L, Shen Q (2020). Idebenone attenuates cerebral inflammatory injury in ischemia and reperfusion via dampening NLRP3 inflammasome activity. Mol Immunol.

[28] Geng X, Li F, Yip J, Peng C, Elmadhoun O, Shen J (2017). Neuroprotection by Chlorpromazine and Promethazine in Severe Transient and Permanent Ischemic Stroke. Mol Neurobiol.

